# Severe hypoxemia caused by right-to-left shunt through a patent foramen ovale associated with right coronary artery malperfusion secondary to acute aortic dissection: a case report

**DOI:** 10.1186/s40981-026-00879-z

**Published:** 2026-11-04

**Authors:** Naoki Takao, Satori Mori, Keiko Takehana, Tomonori Takazawa

**Affiliations:** 1 Department of Anesthesiology, University of Toyama, 2630, Sugitani, Toyama, Toyama 930-0194 Japan; 2 Department of Anesthesiology, Toyama Red Cross Hospital, 2-1-58 Ushijimahonmachi, Toyama, Toyama 930-0859 Japan

**Keywords:** Acute aortic dissection, Coronary artery malperfusion, Patent foramen ovale, Right-to-left shunt, Transesophageal echocardiography

## Abstract

**Background:**

Right coronary artery malperfusion associated with acute type A aortic dissection can cause right ventricular failure. However, hypoxemia due to a right-to-left shunt through a patent foramen ovale secondary to elevated right-sided cardiac pressure has rarely been described in this setting.

**Case presentation:**

An 80-year-old woman with acute type A aortic dissection presented with hypoxemia unexplained by pulmonary disease. Transesophageal echocardiography suggested that right ventricular dysfunction associated with right coronary artery malperfusion had contributed to right-to-left shunting through a patent foramen ovale. Following ascending aortic replacement, she developed hypoxemia after separation from cardiopulmonary bypass. Transesophageal echocardiography showed persistent right ventricular dysfunction and worsening of the shunt. Intraoperative optimization of cardiovascular medications improved hemodynamics, reduced the shunt, and improved oxygenation.

**Conclusions:**

This case suggests that intracardiac right-to-left shunting should be considered in patients with acute aortic dissection and unexplained hypoxemia, and that transesophageal echocardiography may guide perioperative management.

## Background

Coronary artery malperfusion is a life-threatening complication that occurs in 5–13% of patients with Stanford type A acute aortic dissection, and is associated with an in-hospital mortality rate of 18–42% [1, 2]. Right coronary artery malperfusion may cause right ventricular ischemia and subsequent dysfunction. In patients with a patent foramen ovale (PFO), the resulting elevation in right-sided cardiac pressures may produce right-to-left shunting through the PFO and severe hypoxemia [3–5]. We report a case of acute type A aortic dissection, in which right coronary artery malperfusion caused right ventricular dysfunction, leading to clinically significant right-to-left shunting through a PFO and severe hypoxemia.

## Case presentation

An 80-year-old woman presented to the emergency department with dizziness and impaired consciousness. Her medical history included hypertension and chronic atrial fibrillation, for which she was receiving antihypertensive and anticoagulant therapy. On arrival, her blood pressure was 78/48 mmHg, heart rate was 30 beats/min with bradycardic atrial fibrillation, and her oxygen saturation was 91% while breathing 5 L/min oxygen.

**Fig. 1:**
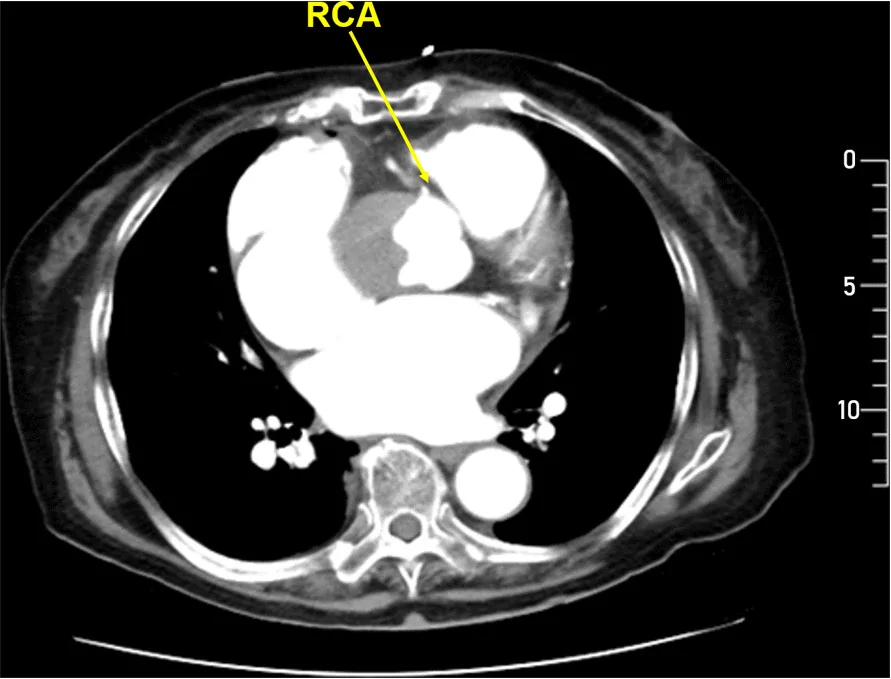
Contrast-enhanced computed tomography findings. The false lumen extended to a point close to the ostium of the right coronary artery. The scale on the right is graduated in centimeters. RCA, right coronary artery

Contrast-enhanced computed tomography revealed Stanford type A acute aortic dissection extending to the aortic root. Although both coronary arteries originated from the true lumen, the false lumen extended close to the right coronary artery ostium (Fig. 1). Electrocardiography showed negative T waves in the precordial leads without ST-segment changes, and her serum CK-MB level was within the normal range. Arterial blood gas analysis showed hypoxemia, with a PaO_2_ of 62.3 mmHg while breathing oxygen at 5 L/min. Chest computed tomography showed no pulmonary parenchymal disease or pulmonary embolism that could explain the hypoxemia.

**Fig. 2:**
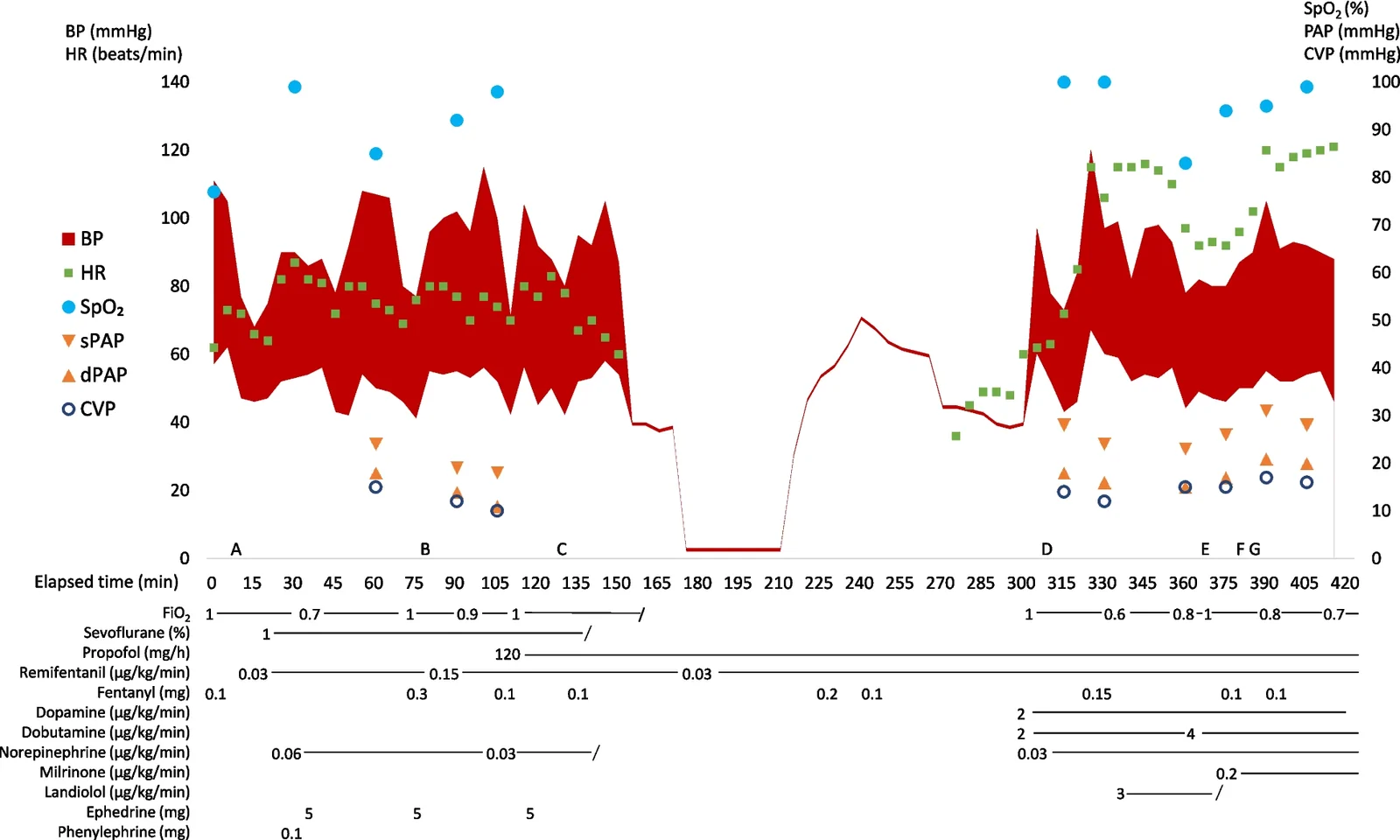
Intraoperative anesthetic record. Severe hypoxemia developed after separation from cardiopulmonary bypass, and improved after optimization of cardiovascular medications. Elapsed time indicates the time since the patient’s arrival to the operating room. BP, blood pressure; HR, heart rate; SpO_2_, peripheral oxygen saturation; sPAP, systolic pulmonary artery pressure; dPAP, diastolic pulmonary artery pressure; CVP, central venous pressure. **A** intubation; **B** start of surgery; **C** start of cardiopulmonary bypass; **D** separation from cardiopulmonary bypass; **E** time point at which the image shown in Fig. 4A was obtained; **F** time point at which the image shown in Fig. 4B was obtained; **G** end of surgery

The patient was transferred to the operating room for surgical repair of the aortic dissection with an arterial pressure line in place. General anesthesia was induced with propofol, succinylcholine, and fentanyl, followed by tracheal intubation (Fig. 2). After transesophageal echocardiography (TEE) probe insertion, a central venous catheter and pulmonary artery catheter were placed via the right internal jugular vein.

After anesthesia induction, her oxygenation deteriorated further, and the PaO_2_/FiO_2_ ratio decreased to approximately 60 (Table 1).

**Table 1:** Intraoperative changes in arterial blood gas parameters

Sampling time (min)	pH	PaO_2_ (mmHg)	PaCO_2_(mmHg)	HCO_3_^−^(mEq/L)	BE (mEq/L)	FiO_2_
20	7.361	66.9	40.7	23.4	−2.4	1.0
110	7.353	63.0	43.9	24.6	−1.1	1.0
140	7.399	285.0	32.7	20.4	−4.6	CPB
210	7.350	279.0	32.6	18.2	−7.7	CPB
325	7.311	275.7	39.2	20.0	−6.5	1.0
360	7.289	46.9	43.8	21.2	−5.6	1.0
390	7.244	64.9	47.8	23.4	−3.3	0.8

Oxygenation improved as the right-to-left shunt through the patent foramen ovale decreased. ‘CPB’ in the FiO_2_ column indicates arterial blood gas sampling was performed during cardiopulmonary bypass.

Her central venous pressure was 15 mmHg, pulmonary artery pressure was 24/18 mmHg, and cardiac index was markedly low at 1.2 L/min/m^2^ (Table 2).

**Table 2:** Intraoperative changes in pulmonary artery catheter–derived parameters

	Before surgical incision (60 min)	At separation from CPB (320 min)	At shunt exacerbation (360 min)	After shunt improvement (390 min)	At ICU admission
CVP (mmHg)	15	14	15	17	16
PAP (mmHg)	24/18	28/18	23/15	31/21	29/17
PAPi	0.4	0.71	0.53	0.59	0.75
CI (L/min/m^2^)	1.2	1.9	1.6	2.2	2.7

Pulmonary artery pulsatility index and cardiac index decreased during exacerbation of the right-to-left shunt, indicating severe right ventricular dysfunction. After optimization of cardiovascular medications between 360 and 375 min, both the pulmonary artery pulsatility index and cardiac index gradually increased.

*CVP* central venous pressure, *PAP* pulmonary artery pressure, *PAPi* pulmonary artery pulsatility index, *CI *cardiac index. PAPi was calculated as (systolic PAP − diastolic PAP)/CVP

**Fig. 3:**
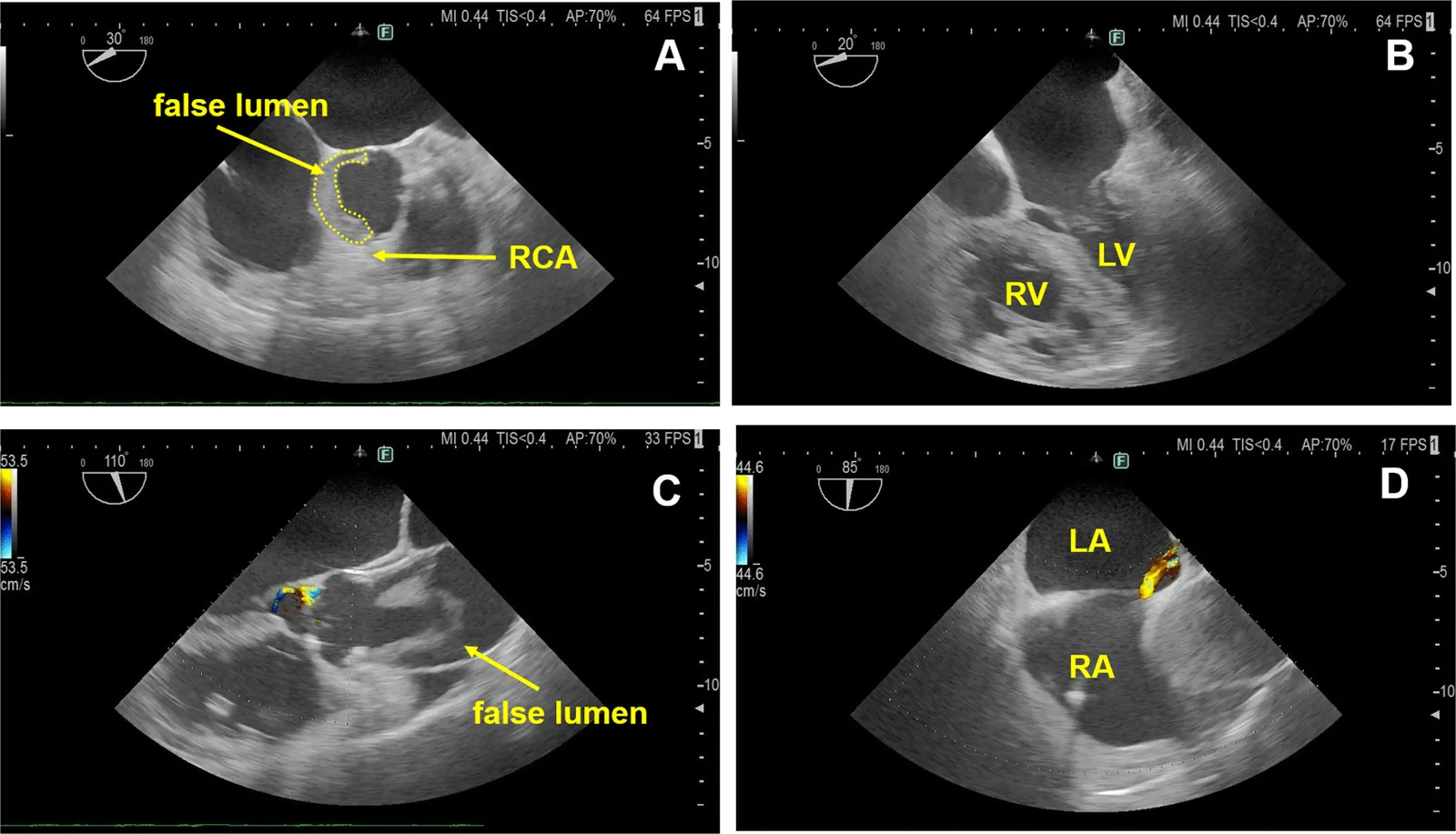
Transesophageal echocardiographic findings after induction of anesthesia. **A** The false lumen compressed the ostium of the right coronary artery. **B** Marked right ventricular dilatation with diffuse hypokinesis was observed. **C** Mild eccentric aortic regurgitation was observed. **D** Right-to-left shunting through a patent foramen ovale was detected. The depth scale on the right side of each panel is graduated in centimeters. RCA, right coronary artery; LV, left ventricle; RV, right ventricle; LA, left atrium; RA, right atrium

TEE findings suggested compression of the right coronary ostium by the false lumen (Fig. 3A). TEE also revealed right ventricular dilatation with diffuse hypokinesis and severe systolic dysfunction, as indicated by a right ventricular fractional area change of 16% (Fig. 3B). Although global left ventricular systolic function was relatively preserved, severe hypokinesis of the inferoseptal and inferior walls was observed. Taken together, these findings suggested right coronary artery malperfusion, although blood flow in the right coronary artery itself could not be directly visualized by TEE. Mild eccentric aortic regurgitation was also observed (Fig. 3C), with no pericardial effusion. Color Doppler echocardiography demonstrated right-to-left shunting through a PFO, which was considered the main cause of hypoxemia (Fig. 3D).

After initiation of cardiopulmonary bypass, the patient was cooled to 25 °C, and circulatory arrest was induced (Fig. 2). Examination showed that the entry tear of the aortic dissection was located near the origin of the brachiocephalic artery, and ascending aortic replacement was performed. Direct surgical inspection confirmed that the right coronary ostium originated from the true lumen, and that the dissection had not extended into the right coronary artery. Based on these findings, closure of the false lumen alone was considered sufficient. Additionally, Doppler assessment of coronary blood flow in the surgical field was not performed during separation from cardiopulmonary bypass.

For myocardial preservation during the surgical procedure, selective antegrade blood cardioplegia was administered through both coronary ostia using a solution prepared from 500 mL of St. Thomas’ Hospital cardioplegic solution No. 2, supplemented with 5 mEq of potassium chloride and blood from the cardiopulmonary bypass circuit. The initial dose was administered at a crystalloid-to-blood ratio of 3:1, followed by repeated doses at a ratio of 1:1 every 30 min. No abnormal increase in perfusion pressure was observed during cardioplegia administration, and prompt myocardial arrest was achieved and maintained throughout aortic cross-clamping. Before release of the aortic cross-clamp, terminal warm blood cardioplegia (hot shot) was administered at a crystalloid-to-blood ratio of 1:3.

After closure of the false lumen and release of the aortic cross-clamp, spontaneous cardiac activity resumed without difficulty. Although PFO closure was considered, it was not performed because the PFO was thought to function as a potential decompression route for the failing right heart.

**Fig. 4:**
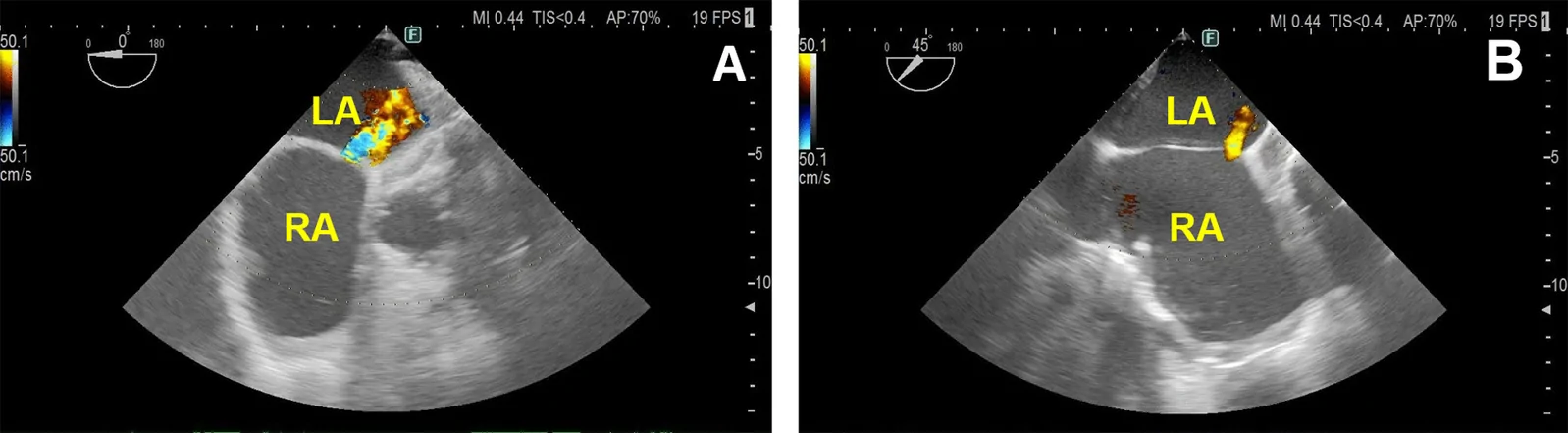
Transesophageal echocardiographic findings after separation from cardiopulmonary bypass. **A** Increased right-to-left shunting through the patent foramen ovale was observed. **B** The right-to-left shunt decreased after optimization of cardiovascular medications. The depth scale on the right side of each panel is graduated in centimeters. LA, left atrium; RA, right atrium

The patient was weaned from cardiopulmonary bypass while receiving dopamine, dobutamine, and noradrenaline. After weaning, her heart rate increased to 115 beats/min, and landiolol administration was started. Subsequently, her oxygenation gradually worsened, and her PaO_2_/FiO_2_ ratio again decreased to approximately 50 (Table 1). TEE showed persistent right ventricular dysfunction and right-to-left shunting through the PFO (Fig. 4A). To improve right ventricular contractility and reduce pulmonary vascular resistance, dobutamine was increased, milrinone was initiated, and landiolol was discontinued. This resulted in improvement in oxygenation, with TEE confirming a reduction in the right-to-left shunt (Fig. 4B) and increase in cardiac index from 1.6 to 2.2 L/min/m^2^ (Table 2).

The patient was postoperatively transferred to the intensive care unit under mechanical ventilation, along with administration of dopamine 5 μg/kg/min, dobutamine 6 μg/kg/min, noradrenaline 0.15 μg/kg/min, and milrinone 0.2 μg/kg/min. Her heart rate was 121 beats/min, blood pressure was 107/64 mmHg, pulmonary artery pressure was 29/17 mmHg, central venous pressure was 16 mmHg, and oxygen saturation was 99% at an FiO_2_ of 0.6. Her CK-MB level peaked at 324.8 ng/mL 3 h after surgery, and subsequently decreased. Although management of right heart failure remained difficult, the patient was extubated on postoperative day 8. Transthoracic echocardiography was performed before extubation, although it was technically limited because of poor image quality, and the interatrial shunt could not be assessed. Repeat transthoracic echocardiography performed after extubation on postoperative day 10, while the patient was still in the ICU, showed reduced wall motion of the right ventricle and the inferior wall of the left ventricle; however, color Doppler imaging showed no detectable shunt through the PFO, and the echocardiographically estimated Qp/Qs ratio was 1.07. No further echocardiographic assessment of the interatrial shunt was performed after discharge from the ICU. The patient underwent rehabilitation and was discharged home with independent ambulation on postoperative day 48.

## Discussion

We report a case of acute aortic dissection, in which right ventricular failure caused by right coronary artery malperfusion led to clinically significant right-to-left shunting through a PFO. Hemodynamic management guided by TEE assessment of right ventricular function and shunt dynamics enabled successful weaning from cardiopulmonary bypass, and resulted in a favorable outcome.

PFO is a common congenital cardiac abnormality, present in approximately 25% of adults [6]. In these patients, increase in right-sided cardiac pressure for any reason may lead to reversal of the pressure gradient between the right and left atria, resulting in a right-to-left shunt through the PFO [7]. Several cases of hypoxemia caused by right-to-left shunting through a PFO associated with right ventricular infarction or right heart failure have been previously reported [3–5]. However, to the best of our knowledge, no previous report has described hypoxemia caused by a right-to-left shunt through a PFO induced by right coronary artery malperfusion associated with acute aortic dissection. Although similar cases have been reported in the perioperative period after surgery for aortic dissection, in those reports, either no intracardiac shunt was identified, or the shunt developed postoperatively due to right atrial compression or morphological changes in the PFO [8–10].

This report highlights the importance of intraoperative TEE in such cases. In the present case, intraoperative TEE contributed to diagnosis, treatment decision-making, and evaluation of the therapeutic response. In surgery for acute aortic dissection, TEE can provide real-time information on the extent of dissection, aortic regurgitation, pericardial effusion, cardiac function, true and false lumen flow, and branch malperfusion [11–13]. It is also useful for diagnosing a right-to-left shunt through a PFO [14]. In the present case, TEE after induction of anesthesia suggested that right ventricular dysfunction associated with right coronary artery malperfusion contributed to the right-to-left shunt.

In acute right heart failure associated with right ventricular infarction, dobutamine may be considered for supporting right ventricular contractility when volume responsiveness is poor and cardiac output is low [15]. Reduction of right ventricular afterload is also important. Milrinone, a phosphodiesterase 3 enzyme inhibitor, has an inotropic effect and can reduce pulmonary vascular resistance, thereby decreasing right ventricular afterload [16]. Pulmonary vascular resistance sometimes increases after cardiopulmonary bypass because of atelectasis, acidosis, inflammatory responses, and endothelial injury [17]. Therefore, in cases such as ours, in which right heart failure and increased pulmonary vascular resistance worsen right-to-left shunting, dobutamine and milrinone may be useful for improving right ventricular function and reducing the shunt.

PFO closure may improve shunt-related hypoxemia; however, it may also eliminate a potential route for right-heart decompression during acute right ventricular failure [4, 7, 18]. Therefore, restoration of coronary perfusion and hemodynamic optimization were prioritized over immediate PFO closure in this patient [4, 5].

This case highlights the importance of considering intracardiac right-to-left shunting in patients with acute aortic dissection and otherwise unexplained hypoxemia. Intraoperative TEE, combined with pulmonary artery catheter-derived hemodynamic assessment, may help elucidate the underlying pathophysiology and guide hemodynamic management in these cases.

## Data Availability

The datasets used and analyzed during the current study are available from the corresponding author upon reasonable request.

## Abbreviations

PFO: Patent foramen ovale
CK-MB: Creatine kinase-myocardial band
PaO_2_: Partial pressure of arterial oxygen
TEE: Transesophageal echocardiography
FiO_2_: Fraction of inspiratory oxygen
PAPi: Pulmonary artery pulsatility index
BP: Blood pressure
HR: Heart rate
SpO_2_: Peripheral oxygen saturation
sPAP: Systolic pulmonary artery pressure
dPAP: Diastolic pulmonary artery pressure
CVP: Central venous pressure
RCA: Right coronary artery
LV: Left ventricle
RV: Right ventricle
LA: Left atrium
RA: Right atrium
